# Case report: Congenital hyperinsulinemia with *ABCC8* gene mutations

**DOI:** 10.3389/fped.2022.914267

**Published:** 2022-08-11

**Authors:** Jin Zhang, Jiyang Wang, Hui Chen

**Affiliations:** ^1^Gansu Women's and Children's Hospital, Lanzhou, China; ^2^Institute of Systems Engineering, Macao University of Science and Technology, Macao, China; ^3^Department of Endocrinology, Lanzhou University Second Hospital, Lanzhou University, Lanzhou, China

**Keywords:** congenital hyperinsulinemia, *ABCC8* gene, subtotal pancreatectomy, diabetes, case report

## Abstract

**Background:**

Congenital hyperinsulinemia (CHI) is an inherited disease of abnormal insulin secretion and is the main cause of persistent and intractable hypoglycemia in infants. The aim of this case report was to investigate the genetic mechanisms and treatment of CHI in an affected patient.

**Case summary:**

We collected clinical data from, and performed gene capture, high-throughput gene sequencing analysis, and Sanger sequencing validation, in a child with CHI and his family to identify the causative gene mutations. Two heterozygous pathogenic mutations in the ATP-binding cassette subfamily C member 8 (*ABCC8*) gene were detected in the child: c.863G>A (p.Trp288Ter) in exon 6 and c.2506C>T (p.Arg836Ter) in exon 21. Sanger sequencing showed that c.863G>A was inherited from heterozygous mutations in the paternal line and c.2506C>T from heterozygous mutations in the maternal line.

**Conclusion:**

The child was a CHI with a biallelic recessive heterozygous mutations in ABCC8 resulting in impairment of its encoded ATP-sensitive potassium (KATP) channel, poor response to diazoxide treatment, and developed diabetes after subtotal pancreatectomy.

## Introduction

Congenital hyperinsulinemia (CHI) is a hereditary disorder of insulin secretion and is the main cause of persistent and refractory hypoglycemia in infants. “congenital hyperinsulinism,” “hyperinsulinemia hypoglycemia of infancy,” “infancy hyperinsulinemia hypoglycemia,” “neonatal hyperinsulinism.” These words above are the names of the different periods of the disease and represent the same meaning. Genetic and molecular biology studies of CHI showed that mutations in the key genes involved in insulin secretion from pancreatic β-cells lead to CHI (1). These genes include *ABCC8, KCNJ11, KCNQ1, CACNA1D, SLC16A1, GLUD1, GCK, HADH, UCP2, HK1, PMM2, PGM1, HNF1A, HNF4A, FOXA2, and EIF2S3* (2, 3). Inactivating mutations in KATP-related genes, including *ABCC8* and *KCNJ11*, are the most common cause of CHI, accounting for approximately 45–50% of cases (4). The treatment of CHI depends on the specific gene mutations involved. We collected clinical data from a 10-year-old male child with CHI and his family. The child developed persistent hypoglycemia at birth and was treated with high concentrations of glucose and diazoxide without satisfactory results. The patient was diagnosed with KATP-associated CHI and underwent subtotal pancreatectomy at the age of 6 months. Subsequently, the patient developed diabetes 4 years after the surgery.

## Case presentation

The child was born at term with a birth weight of 4,400 g. He developed mild asphyxia immediately after birth, for which he remained hospitalized. The child's mother was gravida 2 and para 1 when she was pregnant with the patient. She was healthy during the pregnancy, underwent regular prenatal examinations, and had normal blood glucose and blood pressure levels. At gestation week 36 week, fetal heart rate monitoring revealed absent baseline variation, and the umbilical cord had formed a U-shaped loop around the neck of the fetus. The child was delivered vaginally and had Apgar scores of 6 and 8 at 1 and 5 min, respectively. The child's parents had a non-consanguineous marriage. The patient's maternal and paternal family members had a history of type 2 diabetes.

During admission, the patient has poor ability to respond to external stimuli, other physical examinations showed no abnormality. Hhis fasting blood glucose level was 1.8–2.3 mmol/L (normal: 3.9–6.1 mmol/L), fasting insulin level 25 μIU/mL (normal: 5–20 μIU/mL), and postprandial insulin level 27 μIU/mL (normal: 50–200 μIU/mL). During hypoglycemia, the insulin and C-peptide levels were significantly increased, with the highest recorded insulin level of 182.6 μIU/mL, C-peptide level of 34.97 ng/mL, normal D-hydroxybutyrate level, and free fatty acid level of 0.267 mmol/L. The adrenocorticotropic hormone, cortisol, growth hormone, and thyroid hormone levels were within the normal ranges, and metabolic screening was negative. Abdominal ultrasound revealed a thin pancreas. The patient was administered a high dose of glucose (maximum dose: 10.6 mg/kg/min of 20% glucose) for more than 1 month and intravenous hydrocortisone (maximum dose: 100 mg/m^2^/day) for 17 days. Despite treatment, the blood glucose level remained at 2.4–2.7 mmo1/L; therefore, the patient was diagnosed with CHI. The patient was treated with oral diazoxide at 7.5 mg/kg/day (15 mg every 8 h at the age of 1 month and weight of 6 kg); the diazoxide dose was gradually increased to 12.5 mg/kg/day for 15 days, taken along with hydrochlorothiazide to prevent sodium and water retention. However, the child had a poor response to diazoxide and continued to have frequent hypoglycemia. The patient's parents refused surgical treatment. After 6 months of treatment, the dose of diazoxide was increased to 13.5 mg/kg/day; however, the patient's blood glucose level was still poorly controlled (2.2–3.7 mmo1/L). Therefore, subtotal pancreatectomy was performed at 7 months of age. Histopathological analysis of pancreatic tissue showed areas with an increased islet number and size.

The postoperative level of blood glucose fluctuated between 4.8 and 12.8 mmo1/L. Short-acting insulin was used to control the blood glucose level during the immediate postoperative period. After discontinuation of insulin, dietary control was advised, but the blood glucose level was not monitored. At the age of 4 years, the fasting blood glucose level fluctuated between 3 and 4 mmol/L, whereas the 2-h postprandial blood glucose level fluctuated between 8 and 10 mmol/L; fasting insulin, C-peptide, and glycosylated hemoglobin levels were 1.69 mU/L, 0.46 ng/mL, and 8.9%, respectively. Serum anti-insulin antibodies were not detected. The patient developed postoperative hyperglycemia and was diagnosed with diabetes. Subcutaneous insulin was refused by the family; therefore, the patient was advised to undergo dietary changes for blood glucose control. The 24-h continuous glucose monitoring showed hypoglycemia at approximately 7 AM, with the lowest blood glucose level of 2.2 mmol/L, and the highest blood glucose level (11 mmol/L) at approximately 9 PM. Therefore, 24-h continuous glucose monitoring may be used to monitor the blood glucose levels of children and adjust dietary habits accordingly to reduce blood glucose fluctuation. At the age of 6 years, the patient's blood glucose level was still poorly controlled; the fasting blood glucose level fluctuated between 3.2 and 7.8 mmol/L, the random blood glucose level fluctuated between 6.6 and 11.8 mmol/L, and the fasting and postprandial insulin and C-peptide levels were normal. Stable blood glucose control after insulin therapy. When the patient was 6 years old, his mother became pregnant and underwent amniocentesis that showed no abnormal gene mutations in the fetus. His mother delivered a healthy baby girl after an uneventful full-term pregnancy. At the age of 10 years, the patient's glycosylated hemoglobin level was 6.7%, the fasting blood glucose level fluctuated between 5.49 and 8 mmol/L, the random blood glucose level fluctuated between 5 and 8.34 mmol/L, and continue treatment with insulin. The patient has experienced normal growth and development.

## Outcome

At the age of 4 years, the patient and his parents underwent gene sequencing on DNA isolated from the peripheral serum. First, the DNA was interrupted, and a library was constructed. Then, the DNA of the coding region and adjacent cleavage regions (total target region length: 43401 bp) of the target genes (*ABCC8, KCNJ11, GCK, HADH, INSR, GLUD1, HNF4A, HNF1A, UCP2, FXA2*, and *SLC16A1*) were captured and enriched using a gene chip. A high-throughput sequencing platform was used to detect mutations, and the mutation sites were verified by Sanger sequencing. High-throughput sequencing and Sanger sequencing were performed by Beijing Genomics Institute.

**Figure 1:**
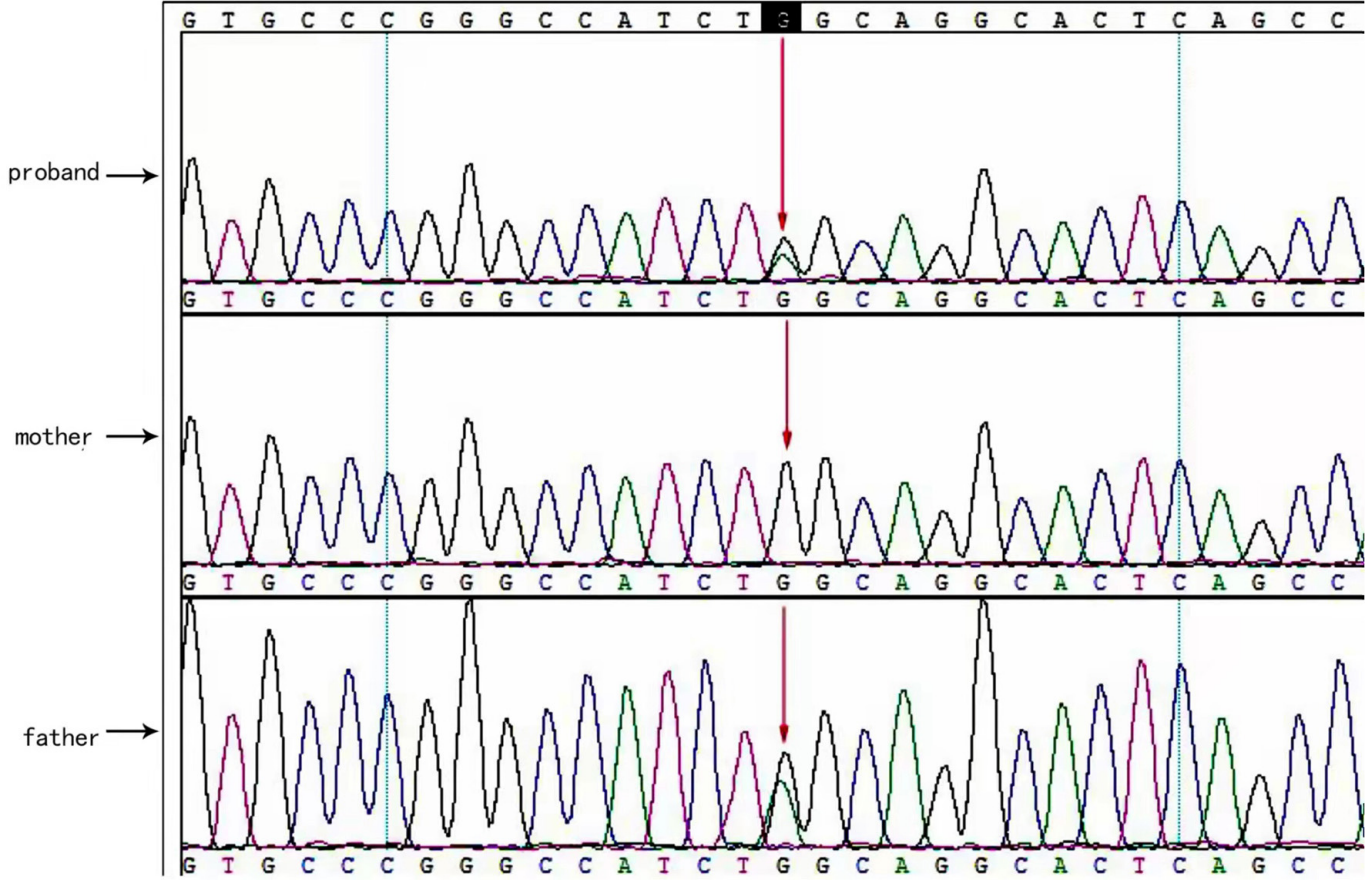
*ABCC8* gene c.863G>A mutated sequence in the proband. Arrows indicate the mutation site.

**Figure 2:**
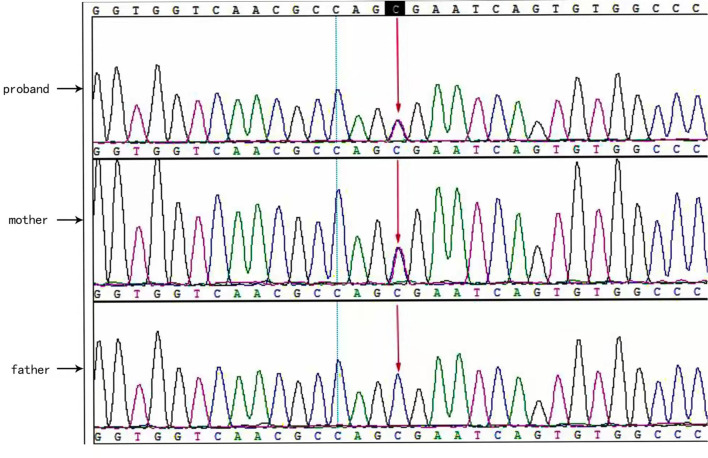
*ABCC8* gene c.2506C>T mutated sequence in the proband. Arrows indicate the mutation site.

**Figure 3:**
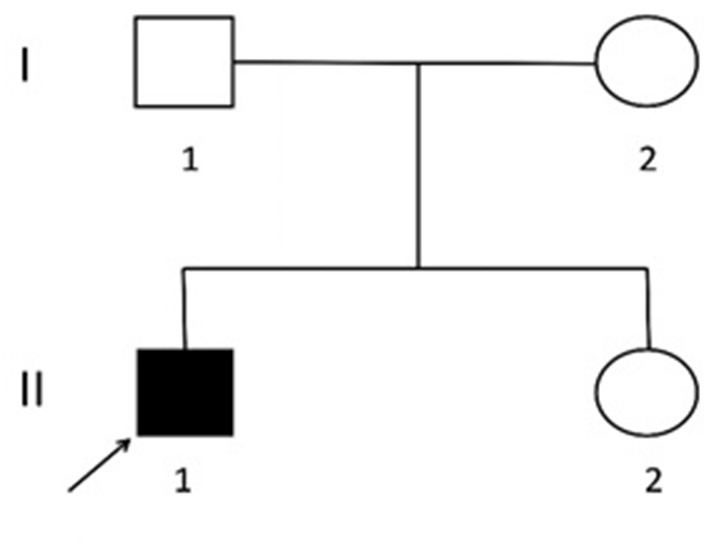
Lineage map. I1. Father: normal phenotype, c.863G>A. I2. Mother: normal phenotype, c.2506C>T. II1. Proband: hypoglycemia, c.863G>A and c.2506C>T. II2. Sister: normal phenotype and genotype.

Two causative mutations in the *ABCC8* gene were detected in the patient (c.863G>A, p.Trp288Ter and c.2506C>T, p.Arg836Ter). Sanger validation detected the c.863G>A mutation in the father (Figure 1) and c.2506C>T in the mather (Figure 2). Therefore, the proband had compound heterozygous mutations in the *ABCC8* gene (i.e., c.863G>A and c.2506C>T). The pedigree of this family is illustrated in Figure 3.

## Discussion

CHI is inherited as an autosomal disorder and is the most important cause of persistent and recurrent hypoglycemia in infants. It has an incidence of 1/50,000 to 1/30,000 in the general population and 1/2,500 in close relatives of affected individuals (5). CHI is characterized by excessive insulin secretion and dysregulation of blood glucose levels, resulting in persistent and refractory hypoglycemia in infants and young children, which may cause irreversible hypoglycemic brain injury (6). Therefore, timely diagnosis and management of CHI are essential. At present, there are no uniform diagnostic criteria for CHI. In 2019, guidelines for the diagnosis and treatment of rare endocrine diseases in China proposed common diagnostic criteria for CHI: in patients with a blood glucose level <3 mmol/L, CHI is diagnosed if insulin and C-peptide levels are measurable, and hypofatty acidemia (plasma level of free fatty acids <1.5 mmol/L) and hypoketonemia (plasma β-hydroxybutyrate level <2 mmol/L) are present. If necessary, a glucagon test can be performed by administering 1 mg intravenous glucagon. Genetic analysis should be performed in suspected patients to guide treatment. For CHI patients, diazoxide therapy is the first-line treatment. Octreotide can be administered as second-line therapy for patients who do not respond to diazoxide. Genetic screening and 18F-L-DOPA-PET/CT are also recommended to guide treatment.

The goal of CHI treatment is to maintain a blood glucose level >70 mg/dL (7). Diazoxide, a KATP channel opener, is effective in CHI patients with intact KATP channels or autosomal dominant KATP mutations (5). KATP mutations accounts for 45–50% of CHI patients, while ~90% of diazoxide-non-responsive patients have mutations in *ABCC8* or *KCNJ11* (8). The therapeutic effect of diazoxide varies significantly among patients according to mutation site. For example, diazoxide is less effective in most CHI patients with mutations in the KATP channel and glucokinase genes; therefore, the use of diazoxide is significantly limited. Octreotide, glucagon, the mTOR inhibitor sirolimus (rapamycin), GDH inhibitors, calcium antagonists, and KATP-channel small-molecular correctors (sulfonylurea and carbamazepine) can be considered for patients who fail to respond to diazoxide. For patients who fail to respond to medical treatment, pancreatectomy should be considered. Paternal monoallelic recessive mutations in the KATP gene have a sensitivity of 84–97% and positive predictive value of up to 94% for predicting focal disease, which can be treated by surgical resection of the lesion (8). Diffuse disease accounts for approximately 60% of cases and affects all pancreatic β-cells (9), and it is caused by biallelic recessive mutations and partial monoallelic dominant mutations in *ABCC8* or *KCNJ11* (7, 10). It usually does not respond to medical treatment and requires subtotal pancreatectomy (excision of 95–98% of pancreas), which often shows unsatisfactory results with a high risk of postoperative diabetes (~50%). In addition, the risk of diabetes increases over time, which necessitates monitoring of pancreatic exocrine secretion. In 2003, the development of 18F-L-DOPA-PET/CT revolutionized the treatment of CHI, as it can accurately localize focal lesions with a sensitivity of 94% and specificity of 100% (8). A previous study (11) reported that before 2009, 91.0% of CHI patients underwent subtotal pancreatectomy in Japan, whereas after 2009, partial pancreatectomy was performed in 64.3% of patients. A preoperative diagnosis of diffuse or focal disease can be made using 18F-L-DOPA-PET/CT, which significantly reduces the number of patients undergoing subtotal pancreatectomy and the risk of diabetes due to unnecessary subtotal pancreatectomy.

In the present case, the proband had macrosomia and developed hypoglycemia immediately after birth. He was treated with high doses of glucose and hydrocortisone; however, his blood glucose level fluctuated between 2.4 and 2.7 mmo1/L (<3 mmol/L), with the highest measured insulin and C-peptide levels of 182.6 μIU/mL and 34.97 ng/mL during hypoglycemia, respectively, blood level of free fatty acids was 0.267 mmol/L (<1.5 mmol/L). In addition, there was no pancreatic space-occupying lesion (e.g., insulinoma); therefore, the patient was diagnosed with CHI and treated with diazoxide for frequent hypoglycemia due to the high likelihood of ATP-sensitive potassium channel mutations. Thereafter, subtotal pancreatectomy was performed, which caused postoperative diabetes (fasting blood glucose >7 mmol/L, glycosylated hemoglobin level >6.5%). Genetic testing was performed at the age of 4 years, which showed compound heterozygous mutations in the *ABCC8* gene (c.863G>A and c.2506C>T). C.863G>A resulted in a switch from tryptophan to a stop codon at position 288 in exon 6. C.2506C>T mutation resulted in a switch from arginine to a stop codon at position 836 in exon 21. Both the c.863G>A and c.2506C>T mutations are nonsense mutation, and introduce stop codons, which prematurely terminate peptide chain synthesis, resulting in fragment deletion and loss of function of the protein. The p.Trp288Ter mutation is located in the transmembrane domain of SUR1 protein, TMD1, which is involved in ATP binding. The p.Arg836Ter mutation is located in the nucleotide-binding domain In review NBD1, which has a high affinity for MgATP and can also sense changes in ATP/ADP concentration and transmit signals to cells, which can cause inactivation of KATP channels after mutation, and then cause excessive release of insulin in the hypoglycemic state. Our patient had autosomal biallelic recessive mutations. These mutations result in severe disease during the neonatal period that responds poorly to diazoxide treatment. The proband's parents and sister did not have hypoglycemic symptoms, but relatives had multiple patients with type 2 diabetes, indicating that the family may have undiscovered islet function problems. The proband's pancreatic beta cells secrete too much insulin, while patients with type 2 diabetes in family members experience insulin hyposecretion or insulin resistance. Mutations in the *ABCC8* gene are also associated with the development of type 2 diabetes, and there may be inheritance of mutations in the *ABCC8* gene in members of this family.

Genetic screening can not only guide the choice of drugs and surgical modalities, but also prenatal and postnatal care. After the diagnosis of CHI, patients should improve the screening of CHI-related causative genes as early as possible. According to the mutations type of pathogenic gene, the efficacy, prognosis and histological type of pancreas of patients can be preliminarily predicted, and combined with the results of 18F-L-DOPA-PET/CT scan, the histological type of pancreas can be more accurately judged to determine whether it is a respectable local lesion. However, genetic screening and 18F-L-DOPA-PET/CT were not commonly used in China before the proband underwent subtotal pancreatectomy. In addition, genetic screening and PET/CT are expensive; therefore, the proband did not undergo genetic screening or 18F-L-DOPA-PET/CT before surgery, and the histology of the pancreas was unclear. As a consequence, diabetes development was inevitable after traditional subtotal pancreatectomy. Due to the above limitations, the patient expressed understanding of our treatment. With the gradual application of genetic testing and PET/CT in China, the number of patients undergoing subtotal pancreatectomy has been greatly reduced, and the number of patients with postoperative complications has also been reduced.

The patient underwent subtotal pancreatectomy in 2011, and the pathological report of the hospital at that time was “increased islet number and volume in some areas of the pancreas.” Several literatures reported that the focal lesion was adenomatous hyperplasia confined to some areas, and the normal structure of the pancreatic lobule was preserved in the lesion, and the surrounding area of the focal lesion was normal; on the other hand, diffuse lesions affect all pancreatic cells and have abnormally enlarged nuclei. According to hospital pathological results, the patient tended to have focal lesions. However, the patient had a biallelic recessive mutation, and many literatures pointed out that this type of mutation was mostly diffuse. Given that the pathological sections of the child had been lost, it was not possible to verify and determine the histological type of pancreas.

## Conclusion

Patients with diffuse *KATP* mutations do not respond to drugs and represent a major challenge for CHI treatment. These patients have poor drug responses and are prone to developing diabetes after subtotal pancreatectomy. The optimal treatment for these patients requires further investigation.

## Data availability statement

The original contributions presented in the study are included in the article/supplementary material, further inquiries can be directed to the corresponding author/s.

## Ethics statement

The studies involving human participants were reviewed and approved by Ethics Committee of the Second Hospital of Lanzhou University. Written informed consent to participate in this study was provided by the participants' legal guardian/next of kin.

## Author contributions

JZ collected data and wrote the manuscript. JW analyzed data. HC contributed to analyze data and revision of the manuscript. All authors contributed to the article and approved the submitted version.

## Funding

This study was supported by Natural Science Foundation of Gansu Province (21JR7RA416), the Second Hospital of Lanzhou University Cuiying Science and Technology Innovation Program (CY2018-ZD02), and Special Fund for Doctoral Training of the Second Hospital of Lanzhou University (YJS-BD-20).

## Conflict of interest

The authors declare that the research was conducted in the absence of any commercial or financial relationships that could be construed as a potential conflict of interest.

## Publisher's note

All claims expressed in this article are solely those of the authors and do not necessarily represent those of their affiliated organizations, or those of the publisher, the editors and the reviewers. Any product that may be evaluated in this article, or claim that may be made by its manufacturer, is not guaranteed or endorsed by the publisher.
